# Community-Engaged Research in Early Home Visiting: A Scoping Review of Peer-Reviewed Literature

**DOI:** 10.1007/s11121-025-01812-z

**Published:** 2025-05-29

**Authors:** Allison West, Diana Eldreth Chute, Jane Daniels, Kelly M. Bower

**Affiliations:** 1 Department of Population Family, & Reproductive Health, Johns Hopkins Bloomberg School of Public Health, 615 N. Wolfe Street, Baltimore, MD 21205 USA; 2 Johns Hopkins School of Nursing, 525 N. Wolfe Street, Baltimore, MD 21205 USA

**Keywords:** Home visitation, Community-engaged research, Scoping review, Health equity

## Abstract

Community-engaged research (CEnR) has potential to advance early home visiting and improve health outcomes for all families by ensuring that research aligns with the needs of the community, methods and procedures are acceptable and accessible, and findings are interpreted accurately and disseminated effectively. We conducted a scoping review to characterize the extent and nature of CEnR in peer-reviewed literature relevant to early home visiting. We searched five scholarly databases for literature published since 2010 describing engagement of community members in research involving evidence-based early home visiting programs. We extracted data on each study’s characteristics, community collaborators, and factors, outcomes, and measures of community engagement. We then coded each study for 16 community engagement components and characterized each study along an established continuum of CEnR. Fourteen articles met all eligibility criteria and were characterized as involving community consultation, community participation, or community-based participatory research. No articles were characterized as community initiated or driven. No studies assessed the impact of community engagement, and only two described barriers or facilitators to engagement. CEnR may be underutilized and underreported in peer-reviewed home visiting research. Findings highlight opportunities to build motivation and capacity for CEnR, transparency in CEnR reporting, and evaluation of CEnR process and impacts.

**Supplementary Information:**

The online version contains supplementary material available at 10.1007/s11121-025-01812-z.

## Introduction

Early home visiting is a voluntary strategy to promote positive outcomes for expectant families and families with young children. Through the federal Maternal, Infant, and Early Childhood Home Visiting (MIECHV) Program and Tribal MIECHV Program, the USA awards grants to 50 states, five territories, the District of Columbia, and numerous tribal communities to implement models that have demonstrated evidence of effectiveness based on HHS criteria (US Department of Health and Human Services, n.d.).

Given its broad dissemination, home visiting has the potential to reduce disparities among families at increased risk for poor health outcomes, yet model impact studies have shown only modest effects on intended outcomes (Michalopoulos et al., 2019; Peacock et al., 2013). Possible explanations for modest effects include differential reach, engagement, and effectiveness for families with varying needs and preferences; unintended variability in implementation; and research designs and methods that provide an incomplete or inaccurate understanding of true program effects. Whereas certain research designs are needed to assess causal impacts of programs on outcomes (e.g., randomized trials and rigorous quasi-experimental designs), a broad range of complementary methods and designs are needed to identify and unpack individual, organizational, community, and systems-level factors that may influence program reach and engagement, implementation fidelity, and outcomes (Brownson et al., 2022). Engaging the communities who stand to be most affected by the findings in the research process is one way to help ensure that a full range of contextual factors are considered and that research findings are relevant, accurate, and useful.

Community-engaged research (CEnR) offers a paradigm for home visiting research to produce generalizable knowledge for action and improve health outcomes for maternal and child health populations through more targeted service delivery (Haroz et al., 2019; Wallerstein et al., 2020). CEnR draws from constructivist and critical theoretical perspectives and emphasizes the value of multiple ways of knowing (Israel et al., 1998). In this article, we use CEnR as an umbrella term to describe an array of research methods that actively engage communities in the research process (Israel et al., 1998). In CEnR, the term “community” refers to groups of people who share common perspectives or interests and who may be affected by the research in positive or negative ways. In home visiting, this may include home visiting recipients or staff, home visiting model purveyors, and funders, to name a few.

Common terms and approaches that fall under the CEnR umbrella include community-based research, community-based participatory research (CBPR), and participatory action research. Although definitions, core principles, and theoretical underpinnings vary across these approaches, all emphasize the role of community in the research process (Brizay et al., 2015). Community engagement in research can be thought of as existing along a continuum (Table 1) (Goodman & Sanders Thompson, 2017; Key et al., 2019; London et al., 2020). Research at one end of the continuum is purely investigator-driven and led; the researcher identifies the research question, selects the design and methods, and interprets and disseminates the results with no input from the community. Research at the other end of the continuum is driven and led by the community (Key et al., 2019). Between the two ends of the continuum are varying types and levels of engagement, and movement from one end to the other is characterized by increases in community involvement, power sharing, and decision-making authority.

**Table 1:** Levels of community engagement defined (adapted from Key et al., 2019)

Level of engagement	Definitions and examples
No community involvement	Researchers work independently. Community is not engaged in any components of the research process
Community informed	Researchers identify the research question(s). Community has a passive role and may not be aware that they are informing research. Information gathered by researchers may inform components of the research, but community is not actively engaged in any components of the research process *Example:* Researchers are interested in maternal depression and attend a community meeting to understand community perspectives on the issue
Community consultation	Researchers identify the research question(s). Community provides limited feedback on one or more components of the research process. Communication is one-way (researchers reach out to the community for feedback) and the community does not have decision-making authority *Example*: Researchers meet with a small group of home visiting clients to seek feedback on an interview guide that will be used in a study about maternal depression
Community participation	Researchers identify the research question(s) and provide opportunities for the community to engage in a defined role in one or more research components. Communication is two-way (researchers to community and community to researchers) and typically ongoing, but community has limited decision-making authority *Example:* Researchers establish a community advisory board that meets regularly to provide guidance on various components of a research study on maternal depression
Community initiated	Community identifies the research question(s) to be addressed but engages researchers to design and implement the research. Community decides whether and how they would like to be engaged, including involvement in research components, level of engagement, and role in decision-making *Example*: Community wants to know if a brief intervention for maternal depression implemented in their community is effective. They contact researchers who design and implement a study. Community lets researchers know they want to be involved in decisions about recruitment and prefer to use existing administrative data to reduce participant burden
Community-based participatory research	Community and researchers identify the research question(s), select the research design and methods, and implement the research project together. Community is equally and equitably engaged in most components of the research. Communication is two-way, and decision-making and ownership of the project and data are shared *Example:* Community and researchers have a longstanding relationship and together identify a research question about maternal depression. In partnership, they select an intervention and design a study based on local knowledge of what would work best for the community
Community driven/led	Community identifies the research question(s) and designs and implements the research. Community seeks the support of the researchers to assist as needed. Community leads the research, owns the data, and holds all decision-making authority *Example*: Community wants to know if an intervention for maternal depression implemented in their community is effective. They design and implement a research study. However, they do not have data analysis skills or software and so hire researchers to assist with this one task

The past three decades have shown increased recognition of the many benefits of CEnR (Balazs & Morello-Frosch, 2013; Greenhalgh et al., 2016; Israel et al., 1998). CEnR is widely recognized as a necessary element to improve overall health outcomes and reduce health disparities because it engages communities in the co-creation, implementation, testing, and translation of viable solutions (Wallerstein & Duran, 2010). Engaging community in intervention development and testing can help ensure that interventions align with needs and preferences of the community, thereby enhancing reach and effectiveness, and, over the long term, health outcomes (Wallerstein, 2021; Wallerstein & Duran, 2010). CEnR also improves the validity, relevance, and use of research by engaging local knowledge, skills, and expertise (Israel et al., 1998). CEnR can help ensure research questions are timely and relevant for a given context, methods and metrics promote participation of all groups, and results are interpreted accurately. Communities that have historically been underserved or experienced barriers to optimum health are also often at highest risk for poor outcomes and the least likely to participate in research; thus, engaging these communities when developing a study can assure fairness, promote trust, and reduce barriers to research participation (Lucero et al., 2020; Occa et al., 2018). Finally, in contrast to extant research paradigms that focus on individual, family, and community deficits, CEnR can help study teams center and build on community strengths and assets (Barkin et al., 2013), while also acknowledging community health factors that may adversely influence health outcomes (Brownson et al., 2022).

In home visiting research, CEnR methods have the potential to strengthen the evidence and better elucidate which home visiting interventions work best, in which contexts, why and how (Home Visiting Applied Research Collaborative, 2022; Supplee & Duggan, 2019), yet the extent to which CEnR methods have been applied and described in published, peer-reviewed home visiting research is unknown. The purpose of this scoping review is to (1) describe the nature and extent of CEnR in home visiting research published in peer-reviewed journal articles and doctoral dissertations, (2) map research studies along a continuum of community engagement, and (3) describe how investigators evaluate the process, outcomes, barriers, and facilitators of CEnR in the home visiting context. Scoping reviews are well suited for characterizing the nature and extent of research on a topic and identifying patterns across the literature (Munn et al., 2018). Our ultimate aim is to advance the use and reporting of CEnR in generalizable research published in scholarly literature.

## Methods

We conducted a scoping review following the approach outlined by Levac and colleagues (2010) and adhering to guidelines set forth in the Preferred Reporting Items for Systematic Reviews and Meta Analyses Extension for Scoping Reviews (PRISMA-ScR) (Tricco et al., 2018). The study team did not register a protocol for this review.

### Search Strategy

**Fig. 1:**
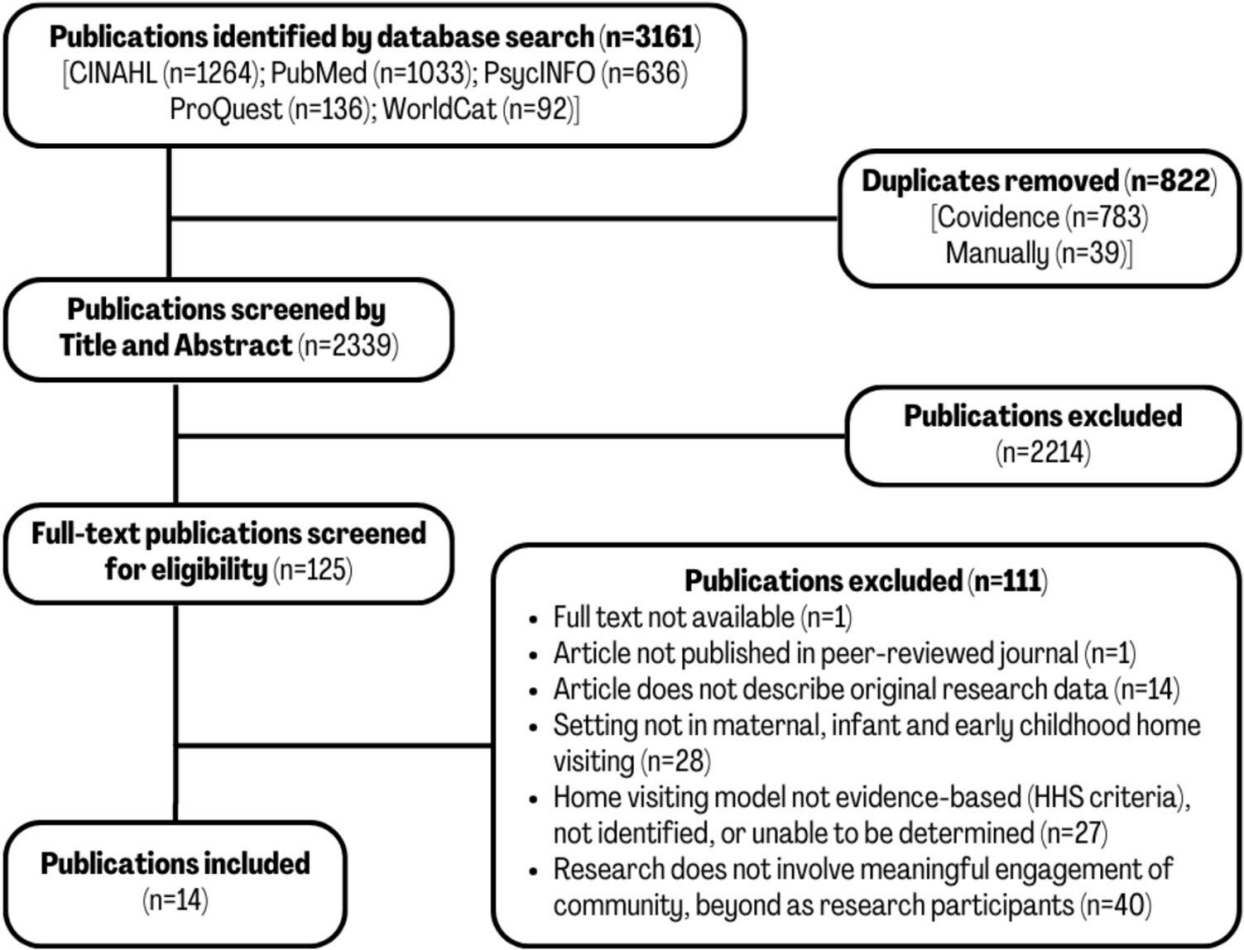
PRISMA flow diagram

We identified studies by searching PubMed, CINAHL, PsycINFO, ProQuest, and WorldCat electronic databases for literature published after MIECHV authorization, between January 1, 2010, and March 13, 2025. We focused on evidence-based home visiting models due to the need for clear inclusion criteria and to distinguish this group of interventions from the larger, more varied universe of interventions that are delivered in the home and/or described as “home visiting.” The search strategy was devised in consultation with a medical library informationist and used terms in three categories: (1) research community collaboration; (2) proper names of 27 home visiting models that met HHS evidentiary criteria, as identified by the Home Visiting Evidence of Effectiveness review (HomVEE, 2024) (see Supplemental Material 1 for list of models); and (3) home visiting (see Supplemental Material 2 for example search terms). This strategy yielded 2339 studies after removal of duplicates (Fig. 1).

### Article Selection

Search results were uploaded into Covidence (2023), web-based software that facilitates systematic reviews. Criteria for article inclusion were (1) published on or after January 1, 2010; (2) published in English; (3) peer-reviewed journal article or dissertation; (4) setting of one of 27 evidence-based home visiting models (HomVEE, 2024); (5) reported using a CEnR approach or method; (6) described original research (e.g., no commentaries, conceptual articles, letters to the editor, reviews); and (7) described meaningful community engagement in planning and implementing the research, beyond research participation.

Article selection was an iterative process because two criteria (6 and 7 above) often could not be confirmed until data were extracted and discussed. For example, we decided at the onset of the study to only include literature that described meaningful community engagement in the research process (criteria #7), which we defined as the presence of one or more community engagement components identified in CEnR literature (detailed below and in Supplemental Material 2) (Brizay et al., 2015; Concannon et al., 2014; Cook, 2008; McFarlane et al., 2022; Spears Johnson et al., 2016). Studies for which no community engagement components were identified were excluded. For example, we excluded articles in which community members served as research participants in focus groups or Delphi panels to inform intervention design or adaptation but did not provide input on the research process. In contrast, we refined criteria #6 (article described original research) after starting eligibility screening; specifically, we decided to exclude articles that focused on quality improvement or program implementation, as these activities typically *required* engagement of program staff or participants in the process.^1^

Each title and abstract were screened by at least two reviewers, one of whom had deep familiarity with home visiting. Conflicts were resolved by consensus. Articles advanced to full-text review if they met inclusion criteria or if a decision could not be made based on the title and abstract alone (*n* = 125). Full texts were reviewed independently by two study staff, and conflicts were discussed and resolved by consensus with the larger study team. If articles linked to supplemental materials or referenced associated articles or reports with additional details on study methods, we included those materials in our assessment.

### Data Charting and Synthesis

Data from eligible articles were extracted independently by two reviewers into a custom form programmed in Covidence. Extraction fields included (1) author and year, (2) country, (3) study aims and methods, (4) home visiting model, (5) community collaborators who were engaged in the research, (6) community engagement components, (7) direction and intensity of engagement, (8) measures used to assess community engagement, (9) any description of the impact of community engagement, (10) barriers and facilitators of community engagement, (11) whether community partners were listed as authors or in acknowledgements, and (12) funding source. We coded each article for 16 community engagement components (inclusion criteria #7) that we drew from lists identified in CEnR literature (Supplemental Material 3) (Brizay et al., 2015; Concannon et al., 2014; Cook, 2008; McFarlane et al., 2022; Spears Johnson et al., 2016). Examples of community engagement components included developing the research design or methods, analyzing data, and interpreting findings.

We then characterized each study along a continuum of community engagement (Key et al., 2019). The continuum describes seven levels of engagement: (1) no community involvement, (2) community informed, (3) community consultation, (4) community participation, (5) community initiated, (6) community-based participatory research, and (7) community driven/led. We developed an initial codebook for characterizing studies a priori based on Key and colleagues’ descriptions of each level. We then refined the codebook using an iterative process to achieve greater clarity and differentiation between levels, taking into consideration the number of community engagement components and the direction and intensity of engagement. The final continuum codebook (Table 1) was applied to each of the eligible articles, and final ratings were determined through discussion and consensus of all study authors. Because study inclusion criteria required evidence of one or more community engagement components, all included studies were characterized as “community consultation” or above on the continuum.

Data synthesis involved grouping articles by how they were rated along the continuum of community engagement. We then sought to characterize the literature within and across groups.

## Results

Fourteen articles met inclusion criteria (Table 2, Supplemental Material 3). Across the 14 articles, eight different evidence-based home visiting models were represented. All but two studies were conducted in the USA. Seven articles described foundational or exploratory descriptive research to better understand home visiting implementation or develop or test research methodologies, four focused on developing and/or pilot testing a home visiting program or intervention, two assessed effectiveness of a home visiting program or intervention, and one described development of a study protocol to develop and test a home visiting program. Two studies used quantitative methods, five used qualitative methods, and seven used a mix of quantitative and qualitative methods.

**Table 2:** Study characteristics and reported community engagement

Primary author (year)	Country and model	Study aim(s) and methods	Community collaborators	Nature of community engagement		
				Community engagement components	Direction and intensity of engagement	Rating of level of engagement
Agu et al. (2021)	USA, MIECHV^a^	Describe the feasibility and utility of implementing Photovoice remotely with HV parents to evaluate factors related to engagement and retention in a perinatal HV program. (QUAL)	Program implementers/stakeholders including MIECHV program directors, supervisors, and administrators	• Conducting needs assessment	Unidirectional communication One reflective process interview per individual and one group debrief	Consultation
Correll et al. (2023)	USA, PAT, HFA, MIHOW	Describe facilitators and barriers to service coordination, to develop a deeper understanding of factors that support service coordination. (QUAL)	Program managers, supervisors, home visitors, and families	• Designing or selecting measures • Interpreting study findings	Uni-directional ND	Consultation
Davis et al. (2016)	USA, PAT^b^	Explore whether home visitor daily practice with families using the Adverse Childhood Experiences framework, focused on individual/family factors, directs attention away from broader social/structural factors within which families are situated. (QUAL)	Home visitors	• Choosing research design and methods	Unidirectional communication 30–45 min interview	Consultation
Kemp et al. (2018)	AUS, MECSH^c^	Describe the protocol for a quasi-experimental study examining the effectiveness of the MECSH program for Aboriginal families in Australia. (QUAN)	Local Aboriginal community representatives and local Aboriginal research and intervention staff	• Intervention design or adaptation • Disseminating findings	Unidirectional communication ND^h^	Consultation
Matone et al. (2018)	USA, EHS^d^, NFP^e^, PAT	Estimate the effectiveness of three MIECHV-funded HV models on early childhood maltreatment. (QUAL/QUAN)	HV leadership	• Choosing research design and methods	Unidirectional communication ND	Consultation
Potter (2017)	USA, HFA	Evaluate the retention rates of families in the HFA program, assess the quality of the home visitor-family relationship, and garner insight into how this relationship impacts overall retention. (QUAL/QUAN)	HFA program management	• Choosing research design and methods	Uni-directional ND	Consultation
Williams et al. (2024)	USA, NFP	Identify a list of process (program implementation) and impact outcomes to inform an evaluation of NFP for expanded populations in Florida. (QUAL/QUAN)	Advisory committee: MCH agencies, HV staff, parent leaders Evaluation team: clinician, researchers, HV staff	• Designing or selecting measures	Uni-directional 3 rounds of data collection via online web surveys and 2 discussion meetings via zoom; committee met monthly via zoom (unknown number of zoom meetings)	Consultation
Folger et al. (2016)	USA, HFA^f^	Describe the development of the community-based enrichment of HV (CBE-HV) approach and evaluate the effectiveness of CBE-HV to strengthen retention and engagement in HV and improve program impacts at the community level. (QUAN)	Community steering committee including leaders of neighborhood councils, faith-based orgs, and orgs devoted to economic development	• Intervention design or adaptation • Recruiting study participants	Bidirectional communication Monthly meetings between community steering committee and HV program	Participation
Oliveira et al. (2022)	UK, VIPP-SD^g^	Adapt the VIPP-SD program to meet the needs of young children with reactive attachment disorder in foster care; conduct a feasibility case series to optimize the treatment manual and assess acceptability of the intervention; assess key stakeholders’ perspectives of implementation issues in preparation for a pilot trial; conduct a pilot RCT of the modified intervention to assess feasibility of a future full-scale trial. (QUAL/QUAN)	Foster carers (foster parents), expert clinicians, and key stakeholders (managers of children’s services, mental health service providers, and foster carers)	• Intervention design or adaptation • Choosing research design and methods • Recruiting study participants	Bidirectional communication ND	Participation
Schumacher (2013)	USA, EHS	Gain an understanding of the experiences and perspectives of EHS program leaders, home visitors, and parents, through individual interviews and home visit observations, to assess what maintains as well as what disrupts engagement for EHS parents and home visitors, to guide better practices for working relationships for EHS interventions. (QUAL)	EHS program director, EHS program manager, home visitors, parents	• Choosing research design and methods • Designing or selecting measures • Interpreting study findings	Bidirectional communication “Hours of informal discussions”	Participation
Stahlschmidt et al. (2018)	USA, PAT	Describe the design, development, implementation, and sustainability of Early Childhood Connections, an innovative program to facilitate coordination between the PAT program and child welfare. (QUAL/QUAN)	Multi-agency workgroup including university-based principal investigator, regional directors from the state child welfare agency, and representatives from HV orgs, early childhood education, special education, childcare associations, health orgs, and “others”	• Conducting needs assessment • Doing background research • Intervention design or adaptation	Bidirectional communication Eight workgroups over a period of 3 years	Participation
Whitesell et al. (2015)	USA, HS/EHS	Assess the need for, feasibility of implementation, and cultural appropriateness of an existing developmental screener, Survey of Well-being of Young Children (SWYC), for use in American Indian and Alaska Native communities. (QUAL)	Members of the SWYC Community of Learning (CoL) including partners in academia, tribal Head Start/Early Head Start, MIECHV^g^, and the Child Care Development Fund	• Intervention design or adaptation • Collecting data • Interpreting study findings	Bidirectional communication Monthly CoL meetings	Participation
Alper et al. (2023)	USA, EHS,HFA	Describe the preliminary assessments of efficacy of Duet, a home-based, early intervention for children 12–24 months, who were at risk for poor language development based on low SES. (QUAL/QUAN)	University-affiliated developmental and clinical scientists and staff of local community organizations (program administrators, directors, and home visitors)	• Writing grant proposal • Choosing research design and methods • Intervention design or adaptation • Implementing the intervention • Interpreting study findings • Disseminating findings	Bidirectional communication ND	CBPR^i^
Mullany et al. (2012)	USA, Family Spirit	Describe the rationale, design, and development of the Family Spirit intervention and the methodology and baseline results of a randomized trial to evaluate its efficacy. (QUAL/QUAN)^b^	Tribal partners in southwestern US, tribal opinion leaders, teen parents, and community board members	• Conducting needs assessment • Intervention design or adaptation • Choosing research design and methods	Bidirectional communication ND	CBPR

Note. ^a^Maternal, Infant, and Early Childhood Home Visiting

^b^Parents As Teachers

^c^Maternal Early Childhood Sustained Home Visiting

^d^Head Start/Early Head Start

^e^Nurse Family Partnership

^f^Healthy Families America

^g^Video-Feedback Intervention to Promote Positive Parenting-Sensitive Discipline

^h^No data

^i^Community-based participatory research

### Nature of Community Engagement in Research

We characterized seven studies as community consultation (Agu et al., 2021; Correll et al., 2023; Davis & Kane, 2016; Kemp et al., 2018; Matone et al., 2018; Potter, 2017; Williams et al., 2024), five as community participation (Folger et al., 2016; Oliveira et al., 2022; Schumacher, 2013; Stahlschmidt et al., 2018; Whitesell et al., 2015), and two as CBPR (Alper et al., 2023; Mullany et al., 2012). No studies met criteria for community-initiated or community led/driven. Across studies, community collaborators ranged from home visiting leadership or staff (*n* = 10), home visiting recipients (*n* = 5), and other community members (*n* = 7).

#### Studies Classified as Community Consultation

Among the seven studies that met criteria for community consultation, researchers consulted with HV leadership and/or staff (Agu et al., 2021; Correll et al., 2023; Davis & Kane, 2016; Kemp et al., 2018; Matone et al., 2018; Potter, 2017; Williams et al., 2024), HV recipients (Correll et al., 2023, Williams et al., 2024), and local community representatives (Kemp et al., 2018; Williams et al., 2024). Intensity of engagement ranged from a single interview (Davis & Kane, 2016), single interview plus a group debrief (Agu et al., 2021), monthly meetings (Williams et al., 2024), or there was no information provided (Correll et al., 2023; Kemp et al., 2018; Matone et al., 2018, Potter, 2017). Five involved consultation on one research component (Agu et al., 2021; Davis & Kane, 2016; Matone et al., 2018, Potter, 2017; Williams et al., 2024), and two involved consultation on two components (Correll et al., 2023; Kemp et al., 2018). Across studies, researchers consulted with community members on choosing research design or methods (*n* = 1), designing or selecting measures (*n* = 4), needs assessment (*n* = 1), intervention design or adaptation (*n* = 1), interpreting findings (*n* = 1), and disseminating findings (*n* = 1).

#### Studies Classified as Community Participation

Of the five studies that met criteria for community participation, three engaged either a Community Workgroup, Steering Committee, or Community of Learning to guide aspects of the research (Folger et al., 2016; Stahlschmidt et al., 2018; Whitesell et al., 2015) and two engaged home visiting recipients and staff (Oliveira et al., 2022, Schumacher, 2013). All but one study (Oliveira et al., 2022) described multiple engagement points. Across the five studies, community collaborators were involved in two or three research components; collaborators were engaged in intervention design or adaptation (*n* = 4), recruiting participants (*n* = 2), interpreting findings (*n* = 2), needs assessment (*n* = 1), background research (*n* = 1), research design or methods (*n* = 2), designing or selecting measures (*n* = 1), and collecting data (*n* = 1). In one study, community collaborators were co-authors, and in another, they were mentioned in study acknowledgements.

#### Studies Classified as Community-Based Participatory Research

Two studies met criteria for CBPR. Mullany et al. (2012) engaged tribal opinion leaders, teen parents, and community board members to design, develop, and test a home visiting program for tribal communities. Alper et al. (2023) engaged home visiting administrators and staff to develop and pilot test an intervention to improve child language development. Between the two studies, collaborators were engaged in seven components of the research process: choosing research design and methods (*n* = 2), intervention design or adaptation (*n* = 2), conducting needs assessment (*n* = 1), writing the grant proposal (*n* = 1), implementing the intervention (*n* = 1), interpreting findings (*n* = 1), and disseminating findings (*n* = 1). Both studies evidenced bidirectional communication and shared decision-making that are characteristic of CBPR. Community collaborators were recognized in acknowledgements in both articles.

#### Synthesis Across All Articles and Levels of Community Engagement

Across all articles, the number of engagement components per study ranged from 1 to 6; in most studies, we identified either one (*n* = 5) or three components (*n* = 5). The components identified most often were intervention design or adaptation (*n* = 7) and needs assessment, designing or selecting measures, and interpreting findings (all *n* = 5). None of the articles reported engaging community collaborators in developing the budget or project management. Seven of the 14 articles specified the number of interactions or duration of engagement with community collaborators; of these, engagement ranged from two interactions over an entire project to monthly meetings for several years. All studies engaged home visiting program staff or community leaders as collaborators; however, only five articles described the engagement of home visiting recipients (e.g., parents or other caregivers) in research components.

### Evaluation of Process, Outcomes, Barriers, and Facilitators to Community Engagement

None of the studies reviewed included measures of process or outcomes of community engagement in the research, although one study (Kemp et al., 2018) discussed broadly how input from the community was important for ensuring feasibility and sustainability in the local context and system. Agu et al. (2021) and Williams et al. (2024) were the only authors to discuss barriers or facilitators to community engagement in the research. Agu described how only program administrators and staff were able to give input because the study design and protocol were preapproved by the study sponsor, whereas Williams described the actions needed to onboard, support, and compensate parent leaders (see also Supplemental Material 4).

## Discussion

This study identified 14 studies published since 2010 that provided unambiguous evidence of CEnR. Consistent with reviews of CEnR in other contexts (Concannon et al., 2014), the roles, activities, and level of engagement of community members varied across articles. Although all articles described at least one component of community engagement, only two studies evidenced active, two-way engagement and shared decision-making across multiple components and stages of the research process that are characteristic of CBPR (Andress et al., 2020). Our results align with other studies that have found variability in how CEnR approaches are described and implemented. For example, Spears Johnson et al. (2016) rated 25 studies characterized by the authors as “CBPR” on 13 research components and found that evidence of actual participation varied widely, with some engaging community in only two components. That only five studies engaged home visiting recipients in CEnR components is notable because research that excludes family perspectives may fail to address the most relevant outcomes, inadvertently reinforce barriers to research participation, and ultimately reduce the utility of the research to improve health outcomes.

There are multiple possible explanations for the small number of CEnR studies identified in this review. One explanation may be that our findings underestimate the true number. We found it difficult to identify meaningful community engagement in many articles, because most articles lacked a clear description of who was engaged in the research, for what purpose, when, and how. This was even the case for some articles that were described by the study authors as using a CEnR approach. Of note, there are many research methods designed to elicit input from community members as *research participants*, such as interviews, Delphi panels, Nominal Group Technique, Photovoice, and Concept Mapping; however, studies that employ these methods are not necessarily CEnR. If researchers alone defined the research questions, developed the methods, carried out the study, and disseminated findings with no input on these processes from the community, we did not characterize a study as CEnR.

Another explanation is that CEnR is underutilized in the home visiting context, which may reflect known barriers for both researchers and communities. For example, factors found to influence front line service providers’ willingness to partner in research include their background, knowledge, skills, attitudes, and expectations regarding research and research partnerships as well as their organizational capacity and resources to take part in research (Pinto et al., 2014). There are also known challenges in IRB review of CEnR proposals, such as community partners not being recognized as members of the research team, required language in consent forms that is inaccessible to partners, and extensive delays in approval processes (Andress et al., 2020; Onakomaiya et al., 2023; Pinto et al., 2014). These factors may affect communication and trust that are central to successful engagement (Han et al., 2021).

Yet another explanation is that CEnR may be less likely to be published in peer-reviewed journals. Publication in peer-reviewed journals may not be community partners’ first choice for dissemination; when communities drive decisions about how findings are shared, they may prioritize products that reach a broader audience of practitioners and policy makers. Further, CEnR may not conform to traditional academic standards of research rigor and quality and is often not rewarded in the promotion and tenure process (Wendling, 2023).

### Implications, Recommendations, and Future Directions

Our difficulty identifying meaningful community engagement in home visiting research underscores the importance of clarity and transparency in reporting (McFarlane et al., 2022). Fortunately, there are several useful guidelines, standardized rubrics, and other tools available to support greater transparency (Concannon et al., 2014; Harrison et al., 2019; Kato et al., 2020; Smith et al., 2010). We recognize that journals often impose strict word and character limits, yet we contend that key information can be conveyed clearly and concisely. This can be accomplished, in part, with an explicit statement describing who was engaged in the research process, when, how, and for what purpose, such as in this fabricated example:

Community research partners were 5 home visitors and 2 supervisors from 3 Baby Hugs home visiting programs in Georgia [WHO] who met with researchers monthly for one year [WHEN] and were engaged in conceptualizing the study, refining recruitment materials, selecting some measures, interpreting results, and writing this manuscript and a research-to-practice brief that was shared via newsletter with the state home visiting network [HOW]. See supplemental materials for more details on community partners and engagement [WHERE TO FIND MORE INFORMATION].

To address barriers to conducting CEnR, research partners can draw from extensive literature describing best practices in CEnR, both generally and with specific populations highly relevant to early home visiting, such as families with children (Jacquez et al., 2013) and underserved groups (De Las Nueces et al., 2012; McElfish et al., 2019). Other useful resources include tools and training to support engaging home visiting staff and other partners in home visiting research (Andres et al., 2020; Around Him & Kane, 2022; HARC, 2023). Finally, London et al. (2020) offer a framework for designing effective CEnR that considers alignment between the research problem, the capacities and resources of the researchers and the community, and the broader sociopolitical context.

That no studies measured the process or outcomes of CEnR is also noteworthy and aligns with findings from other fields (Concannon et al., 2014; Goodman & Sanders Thompson, 2017). Advances in measurement development are promising; tools exist for assessing influential factors, process indicators, and outputs of CEnR (Esmail et al., 2015; Israel et al., 2013). Recently, Luger et al. (2020) developed a list of 69 measures that assess CEnR context, process, and outcomes, and the list continues to grow (Boursaw et al., 2021).

This study has some limitations. This review was narrowly focused on understanding the nature and extent of community engagement in original research as described in peer-reviewed, published literature. We did not include unpublished research or evaluation, where CEnR may be more prevalent. Of note, although meaningful community engagement was one of our inclusion criteria, identifying this in articles was much more difficult than expected because most articles lacked sufficient detail regarding CEnR methods. Consequently, our results may underestimate the true number of CEnR studies. Similarly, although a strength of the study was the use of a framework to identify and characterize CEnR, we may have mischaracterized studies that lacked sufficient methodological details. Because this review was limited to evidence-based home visiting models as defined by HHS criteria, findings may not generalize to the broader array of home visiting models. We may have also missed studies that incorporate CEnR in building evidence for new or emerging home visiting models and cross-model research that did not name a specific home visiting model. Finally, as the purpose of the scoping review was to assess the nature and extent of relevant research, we did not assess the quality of included studies.

## Conclusions

There is a strong rationale for CEnR in home visiting, where there is growing recognition of the need to include and amplify community voices in research, practice, and policy. The nature and extent of CEnR in home visiting remain unclear based on the results of this review. It may be that challenges with engagement persist, authors’ descriptions of methods are incomplete, or CEnR is not published in scholarly outlets. Inadequate representation and engagement of families and other community members in home visiting research may reduce the validity, relevance, usefulness, and translation of research findings to practice and further contribute to poorer health outcomes over time. The field of home visiting may benefit from further critical examination of current research practices and intentional promotion and integration of CEnR.

## Supplementary Information

Below is the link to the electronic supplementary material.

Supplementary file1 (DOCX 28 KB)

## Data Availability

NA.

## Footnotes

1 We verified articles describing quality improvement by checking for human subjects research review and approval.
