# Supplementary material for: Community-Engaged Research in Early Home Visiting: A Scoping Review of Peer-Reviewed Literature
Source: Prev Sci. 2025 May 29;26(5):703–15. doi: 10.1007/s11121-025-01812-z (PMC12245935; doi:10.1007/s11121-025-01812-z)
Supplement: Supplementary file 1 — Supplementary file1 (DOCX 28 KB) [file 11121_2025_1812_MOESM1_ESM.docx]

**Supplemental Material 1**

*Eligible Home Visiting Models Based on HHS Evidentiary Criteria (HomVEE, 2024)*

| Home Visiting Model |
| --- |
| 1. Attachment and Biobehavioral Catch-Up (ABC) -Infant |
| 1. Child First |
| 1. Early Head Start Home-based option (EHS) |
| 1. Early Intervention Program for Adolescent Mothers (EIPAM) |
| 1. Early Start |
| 1. Family Check-Up® For Children |
| 1. Family Connects |
| 1. Family Spirit® |
| 1. Health Access Nurturing Development Services (HANDS) Program |
| 1. Healthy Beginnings |
| 1. Healthy Families America (HFA)® |
| 1. Healthy Steps (National Evaluation 1996 Protocol) |
| 1. Home Instruction for Parents of Preschool Youngsters (HIPPY)® |
| 1. Intervention Nurses Start Infants Growing on Healthy Trajectories (INSIGHT) |
| 1. Maternal Early Childhood Sustained Home-Visiting Program (MECSH) |
| 1. Maternal Infant Health Outreach Worker (MIHOW)® |
| 1. Maternal Infant Health Program (MIHP) |
| 1. Minding the Baby® Home Visiting (MTB-HV) |
| 1. Nurse-Family Partnership (NFP)® |
| 1. Oklahoma’s Community-Based Family Resource and Support (CBFRS) Program |
| 1. Parents as Teachers (PAT)® |
| 1. Play and Learning Strategies (PALS) Infant |
| 1. Preparing for Life—Home Visiting |
| 1. Promoting First Relationships® Home Visiting Intervention Model (PFR) |
| 1. SafeCare Augmented |
| 1. Video-Feedback Intervention to promote Positive Parenting (VIPP) |
| 1. Video-Feedback Intervention to promote Positive Parenting – Sensitive Discipline - (VIPP-SD) |

**Supplemental Material 2**

*Example Search Strategy - PubMed*

| Search concept | Search terms |
| --- | --- |
| #1 Research community collaboration | "Community-Based Participatory Research"[Mesh] OR "Community Participation"[Mesh] OR **"Public-Private Sector Partnerships"[Mesh]** OR "Stakeholder Participation"[Mesh] OR "Patient Participation"[Mesh] OR "community-engaged research*"[tw] OR "community-based participatory research*"[tw] OR "participatory research*"[tw] OR "participatory action research*"[tw] OR "community-based research*"[tw] OR "community-academic partner*"[tw] OR "research community partner*"[tw] OR "research community collaboration"[tiab: ~5] OR "research community collaborations"[tiab: ~5] OR "research practice partner*"[tw] OR "research practice collabor*"[tw] OR "academic community partner*"[tw] OR "academic community collabor*"[tw] OR "academic practice partner*"[tw] OR "academic practice collabor*"[tw] OR "university community collabor*"[tw] OR "university community partnership*"[tw] OR "university practice collaboration"[tiab: ~5] OR "university practice collaborations"[tiab: ~5] OR "university practice partnership"[tiab: ~5] OR "university practice partnerships"[tiab: ~5] OR "community participatory research*"[tw] OR "community collaborative research*"[tw] OR "community partnered research*"[tw] OR "community engage*"[tw] OR "action research*"[tw] OR "community empowerment research*"[tw] OR "university collabor"[tiab: ~5] OR "university partner"[tiab:~5] OR “stakeholder*”[tw] OR “stakeholder engag*”[tw] OR “stakeholder engagement”[tiab: ~5] OR “stakeholder engagements”[tiab: ~5] OR “stakeholder participat*”[tw] OR "stakeholder involv*"[tw] OR "patient engage*"[tw] OR “patient engagement”[tiab:~5] OR “patient engagements”[tiab:~5] OR "patient participat*"[tw] OR "patient involv*"[tw] |
| #2 Evidence-based home visiting models (identified as such by HomVEE) | “Attachment and Biobehavioral Catch-Up*”[tw] OR “Attachment and Biobehavioral Catch Up*”[tw] OR “Child First*”[tw] OR “Early Head Start*”[tw] OR “Early Intervention Program for Adolescent Mother”[tiab: ~5] OR “Early Intervention Program for Adolescent Mothers”[tiab: ~5] OR “Early Start*”[tw] OR “Family Check-Up for Children”[tiab: ~5] OR “Family Check Up for Children”[tiab: ~5] OR “Family Connect*”[tw] OR “Family Spirit*”[tw] OR “Health Access Nurturing Development Services”[tiab: ~5] OR “Health Access Nurturing Development Service”[tiab: ~5] OR “Healthy Beginning*”[tw] OR “Healthy Families America*”[tw] OR “Healthy Step*”[tw] OR “Home Instruction for Parents of Preschool Youngsters”[tiab: ~5] OR “Home Instruction for Parents of Preschool Youngster”[tiab: ~5] OR “Intervention Nurses Start Infants Growing on Healthy Trajectories”[tiab: ~5] OR “Maternal Early Childhood Sustained Home-Visiting Program”[tiab: ~5] OR “Maternal Early Childhood Sustained Home Visiting Program”[tiab: ~5] OR “Maternal Infant Health Outreach Worker*”[tw] OR “Maternal Infant Health Program*”[tw] OR “Minding the Baby*”[tw] OR “Nurse Family Partnership*”[tw] OR “Oklahoma’s Community-Based Family Resource and Support Program”[tiab: ~5] OR “Oklahoma’s Community Based Family Resource and Support Program”[tiab: ~5] OR “Parents as Teacher*”[tw] OR “Play and Learning Strategies”[tiab: ~5] OR “Promoting First Relationships Home Visiting Promotion Model”[tiab: ~5] OR “Promoting First Relationship*”[tw] OR “SafeCare*”[tw] OR “Video-Feedback Intervention to Promote Positive Parenting-Sensitive Discipline”[tiab: ~5] OR “Video Feedback Intervention to Promote Positive Parenting Sensitive Discipline”[tiab: ~5] OR “Video-Feedback Intervention to Promote Positive Parenting”[tiab: ~5] OR “Video Feedback Intervention to Promote Positive Parenting”[tiab: ~5] |
| #3 Home visiting | "House Calls"[Mesh] OR "home visit*"[tw] OR "homevisit*"[tw] OR "MIECHV"[tw] OR "in-home visit*"[tw] OR “home-based*”[tw] OR “home-visit*”[tw] |
| #4 Search | #1 AND (#2 OR #3) |

**Supplemental Material 3**

| *Community Engagement Components* |
| --- |
| 1. Conducting needs assessment |
| 1. Writing funding proposal |
| 1. Developing budget |
| 1. Conducting background research |
| 1. Choosing research design and methods |
| 1. Developing sampling procedures |
| 1. Designing or selecting measures |
| 1. Intervention design or adaptation |
| 1. Recruiting study participants |
| 1. Implementing the intervention |
| 1. Collecting data |
| 1. Analyzing data |
| 1. Interpreting study findings |
| 1. Publishing findings in formal reports or journals |
| 1. Disseminating findings to broad audiences |
| 1. Project management |

**Supplemental Material 4**

*Study Data Extracted for Measures, Impact, Facilitators, Barriers, Authorship, and Funding Source*

| Primary Author (year) | Measures  of Community Engagement | Impact of Community Engagement | Facilitators of Community Engagement | Barriers to Community Engagement | Community Partners as Authors or in Acknowledgements | Funding Source(s) |
| --- | --- | --- | --- | --- | --- | --- |
| Agu et al. (2021) | ND | ND | ND | Study protocol was preapproved by funder, thus only program administrators/staff could give input. | No | Florida Maternal, Infant, and Early Childhood Home Visiting Initiative, Health Resources and Service Administration (HRSA) of the U.S. Department of Health and Human Services |
| Correll et al.  (2023) | ND | ND | ND | ND | No | Robert Wood Johnson Foundation and the Home Visiting Applied Research Collaborative (HARC) |
| Davis et al. (2016) | ND | ND | ND | ND | Acknowledgements | Two internal grants from Bates College |
| Kemp et al. (2018) | ND | ND | ND | ND | Acknowledgements | Australian National Health and Medical Research Council; CAGES Foundation and the Vincent Fairfax Family Foundation |
| Matone et al. (2018) | ND | ND | ND | ND | No | Department of Human Services, Commonwealth of Pennsylvania |
| Potter (2017) | ND | ND | ND | ND | Acknowledgements | Not specified |
| Williams (2024) | ND | ND | Parent leaders received specialized onboarding, training in basic research concepts, technology support in the form of a tablet or webcam, and compensation for their time (monthly stipend) | ND | Yes | Nurse-Family Partnership® (NFP) National Service Office |
| Folger et al. (2016) | ND | ND | ND | ND | No | US National Institute of Mental Health (NIMH) |
| Oliveira et al. (2022) | ND | ND | ND | ND | Acknowledgements | National Institute for Health and Care Research (NIHR) Health Technology Assessment programme |
| Schumacher (2013) | ND | ND | ND | ND | No | Not specified |
| Stahlschmidt et al. (2018) | ND | ND | ND | ND | No | US NIMH & NIMH T32 |
| Whitesell et al. (2015) | ND | ND | ND | ND | Authors | US Administration for Children and Families Office of Planning Research and Evaluation |
|  |  |  |  |  |  |  |
| Alper et al. (2023) | ND | ND | ND | ND | Acknowledgements | William Penn Foundation and the Bezos Family Foundation |
| Mullany et al. (2012) | ND | ND | ND | ND | Acknowledgements | US National Institute on Drug Abuse |

*Note.* ND = No data.
